# A nursing perspective on human-AI collaboration in personalized breast cancer care pathways

**DOI:** 10.3389/fonc.2026.1784401

**Published:** 2026-03-11

**Authors:** Jia-xin Zhang, Xue Zhao, Ji-hong Tao, De-chun Su

**Affiliations:** 1 Second Ward of General Surgery Department, Hongqi Hospital Affiliated to Mudanjiang Medical University, Mudanjiang, China; 2 Department of Operating Room, Hongqi Hospital Affiliated to Mudanjiang Medical University, Mudanjiang, China

**Keywords:** artificial intelligence, breast cancer, digital health, human-machine collaboration, nursing perspective, patient-centered care

## Abstract

The integration of artificial intelligence (AI) has shown strong performance in well-defined clinical tasks, particularly in reader studies and workflow simulations. Translation into routine clinical environments, however, depends on local integration strategies, threshold selection, and governance arrangements. This perspective article adopts a nursing science perspective to argue that human-AI collaboration represents more than a technological addition—it constitutes a fundamental shift toward a synergistically enhanced nursing practice. Central to this paradigm is the effective integration of nursing expertise with algorithmic capabilities throughout all stages of care. When appropriately implemented and supervised, such integration has the potential to enhance both precision and efficiency in nursing practice. Importantly, it should be carried out in a way that preserves core nursing values, including patient-centered care, respect for individual dignity, and the integrity of therapeutic relationships. The proposed framework establishes a conceptual foundation intended to support the design of ethically aligned and clinically relevant human-AI systems. It further aims to guide the evolution of nursing practice within personalized breast cancer care.

## 1 Introduction

Using GLOBOCAN 2022 estimates, breast cancer is the most commonly diagnosed cancer in women worldwide (1). Advances in diagnosis and treatment have significantly extended patient survival, shifting the focus of disease management from acute-phase treatment to comprehensive, long-term care (2). This continuum spans prevention, screening, treatment, rehabilitation, and survivorship support. Consequently, care delivery now faces several complex challenges: the biological heterogeneity of breast cancer demands highly individualized management; prolonged survival introduces chronic needs, including symptom control, psychological distress, and recurrence monitoring; and patients, families, and healthcare systems experience substantial physical, emotional, and economic burdens (3–5). These factors collectively underscore an urgent need for more personalized and precise models of care.

In response, the integration of advanced technologies, particularly artificial intelligence (AI), has become a pivotal trend. AI technologies are increasingly being evaluated across the care spectrum—from risk assessment and imaging support to treatment planning and prognostic estimation. At present, the strongest evidence supports their use in well-defined, bounded tasks under controlled settings, while effectiveness in routine clinical practice remains dependent on integration approaches, oversight frameworks, and institutional governance (6–8). Such tools are intended to enhance clinical precision, improve decision-making efficiency, and potentially support resource allocation, depending on local implementation conditions (9).

However, current research remains predominantly rooted in technical or medical perspectives, focusing on algorithmic performance or clinical validation (10, 11). A critical gap exists in examining how human-machine collaboration fundamentally reshapes nursing practice, which lies at the heart of patient care. Nursing science, as the discipline central to sustained and holistic care delivery, should address key questions: How does AI integration reconfigure nursing workflows and responsibilities? How does it affect the therapeutic nurse-patient relationship? And what implications does it hold for the evolving identity, expertise, and values of the nursing profession? Ignoring these questions may lead to technologically advanced but humanistically misaligned care systems.

For the purposes of implementation and governance, this perspective article adopts a functional definition of human-AI collaboration. We identify several recurrent categories of AI participation within nursing workflows: (1) Decision support, where systems generate options or risk estimates while clinicians retain interpretive authority; (2) Triage and prioritization, where algorithms flag urgency or direct attention; (3) Longitudinal monitoring, for detecting deviations or trends over time; (4) Documentation assistance, where information is summarized or structured; (5) Patient-facing support, such as reminders or navigation prompts; and (6) Selective automation, where predefined steps are executed under human supervision. These categories differ in their risk exposure, oversight needs, and accountability structures. A detailed mapping of these functional categories and their distribution across care stages is provided in **Supplementary Table 1**.

**Figure 1:**
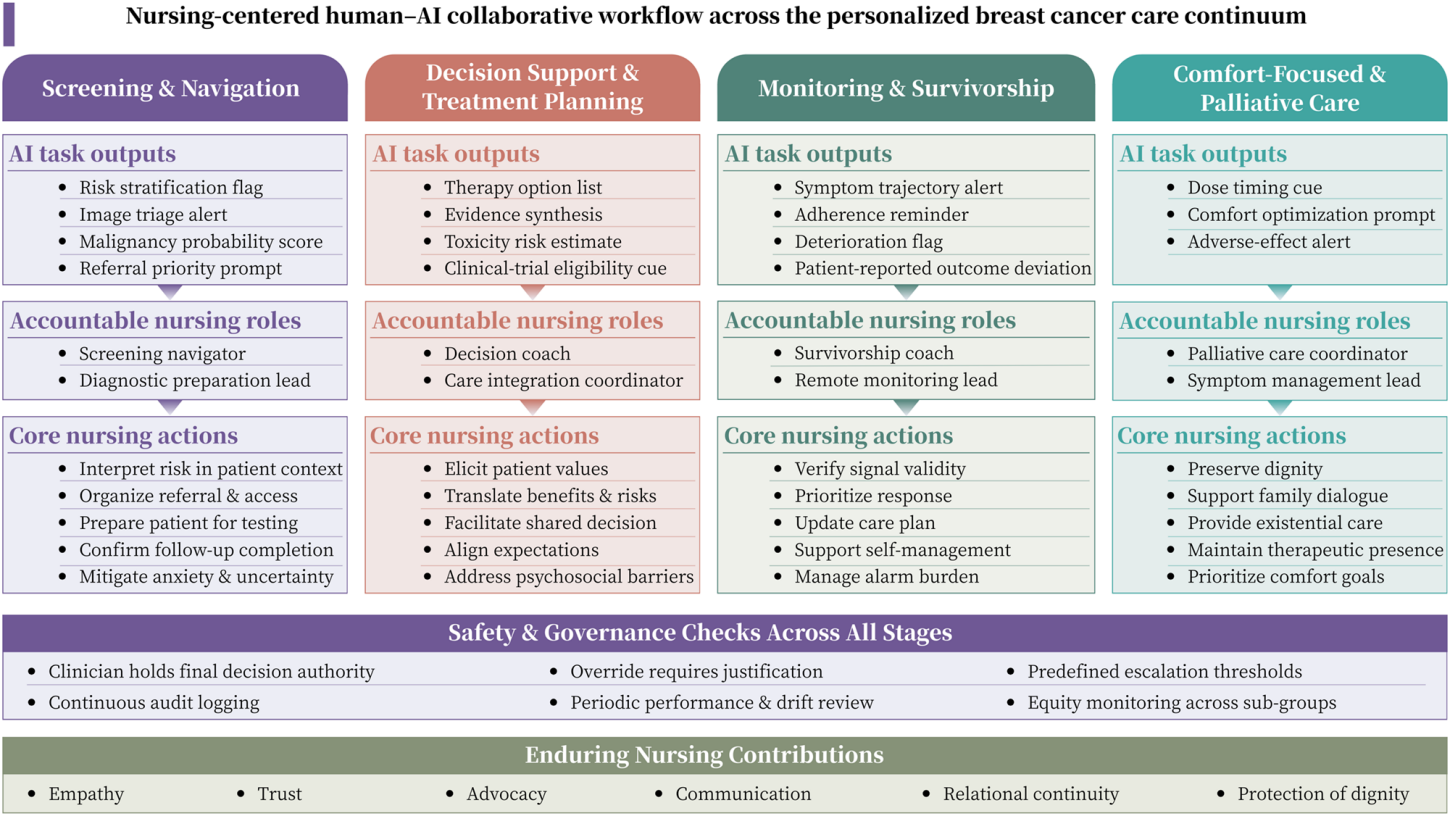
Nursing-centered human-AI collaborative workflow across the personalized breast cancer care continuum. This figure presents stage-specific AI outputs and accountable nursing roles from screening to palliative care. It highlights embedded governance-including decision authority, escalation rules, and equity monitoring and workflow transitions, while affirming the enduring human contributions essential to technology-enhanced practice.

Therefore, this perspective article proposes a nursing-centered analytical framework to examine human-AI collaboration within personalized breast cancer care pathways (**Figure 1**). It aims to clarify the operational paradigms, identify essential components, investigate practical challenges, and suggest future directions for implementation. By bridging the gap between technological innovation and the core tenets of nursing practice, this work seeks to inform the development of ethically sound, effective, and person-centered intelligent care models.

## 2 Core nursing elements of personalized breast cancer care pathways

A personalized care pathway for breast cancer should be rooted in the distinct perspective of nursing science, guided by essential professional principles. These principles collectively establish a comprehensive framework for care delivery, ensuring it addresses both the biomedical complexity of the disease and the comprehensive, multidimensional needs of the individual.

### 2.1 Continuity across the entire care continuum

A personalized pathway should encompass the full trajectory of the illness. It should facilitate seamless transitions from risk assessment and screening through diagnosis, treatment, and rehabilitation, to long-term survivorship or palliative care (12, 13). This approach moves beyond episodic, fragmented models by emphasizing proactive coordination between care stages (14). The aim is to ensure sustained nursing engagement, enabling patients to receive consistent and timely professional support at every phase of their health journey. Such continuity supports effective management of chronic symptoms, monitoring of long-term effects, and enhancement of overall quality of life (13, 15).

### 2.2 Integration of multidimensional needs

Breast cancer affects patients beyond the physical dimension, influencing psychological, social, and spiritual well-being (16). An effective care pathway should therefore integrate multiple layers of need: precise management of physical symptoms, such as pain, fatigue, and treatment-related side effects; structured psychosocial support to address emotional and relational challenges; tailored health communication and decision aids to promote understanding and autonomy; and attention to spiritual concerns related to meaning and coping (17–19). Achieving this integration requires holistic assessment methods and collaborative interprofessional teamwork.

### 2.3 Dynamic adaptability

Personalized care is an iterative process, not a fixed protocol (20). The care plan should remain flexible and responsive to changes in the patient’s condition, treatment response, personal values, and life circumstances (21, 22). This requires ongoing, structured reassessment to ensure the pathway evolves in alignment with the patient’s current health status and lived experience—a fundamental aspect of truly patient-centered care (23, 24).

### 2.4 Empowerment and shared participation

Contemporary care emphasizes enabling patients and families to actively participate in health management. Nursing plays a pivotal role in fostering self-efficacy through education, skill development, and resource facilitation (25). Equally important is promoting shared decision-making, where patients are supported as informed partners in choosing treatments and defining care goals (26). This empowerment approach enhances adherence, improves outcomes, and helps individuals regain agency, reflecting a holistic and humanistic model of care (27).

In summary, continuity, integration, adaptability, and empowerment are interdependent elements that form the foundation of personalized breast cancer care pathways. Together, they provide a coherent framework for examining how human and technological collaboration can meaningfully enhance care delivery.

## 3 Specific forms of human-machine collaboration across care pathway stages and the reshaping of nursing roles

In personalized breast cancer care pathways, human-machine collaboration is not a single static model, but rather a set of evolving practices that change across different stages of the disease (**Figure 1**). When implemented within validated frameworks and supported by appropriate accountability structures, these emerging forms of collaboration may contribute to shifts in nursing responsibilities. Specifically, nurses are transitioning from a primarily task-oriented approach toward a role characterized by greater integration of information, nuanced interpretation, and a strengthened emphasis on humanistic caregiving. The operational responsibilities, decision authority, safeguards, and evaluation metrics associated with these collaborations are summarized in **Table 1**. Importantly, all stages of the clinical pathway, nurses remain responsible for communicating uncertainty, clarifying model limitations, and ensuring that algorithmic outputs are applied strictly within their validated and appropriate clinical contexts.

**Table 1:** Operational Framework for Human–AI Collaboration Across the Breast Cancer Care Pathway.

Care Stage	AI Task Outputs	Nursing Role	Decision Authority	Safeguards & Override Logic	Equity Checks	Evaluation Indicators
Screening & Diagnosis	Risk score; image triage flag; reader support visualization	Nurse navigator/ risk communicator	Clinician holds final responsibility; AI advisory	Override required when clinical assessment disagrees; document AI output, rationale, patient context; escalate if high-risk or low-confidence flag	Monitor referral rates and false positives across age, access status, geography	Time to diagnosis; navigation completion; inappropriate referral rate
Treatment Planning & Delivery	Therapy option list; toxicity risk estimate; interaction alerts	Decision coach/care coordinator	Shared decision with oncologist; nurse ensures contextualization	Escalate when patient values conflict with ranking; record deviations from AI suggestions	Stratify recommendations & outcomes by comorbidity and vulnerability markers	Decisional conflict; adherence; preventable toxicity events
Rehabilitation & Survivorship	Symptom trend alerts; deterioration flag; adherence reminder	Health coach/longitudinal monitor	Nurse interprets; physician review for escalation	Mandatory review for sustained alerts; filter alarm fatigue; maintain audit trail	Compare alert responsiveness by age, digital literacy, rurality	Readmission; symptom control; sustained engagement
Advanced/ Palliative Care	Comfort parameter prompts; medication timing support; risk alerts	Palliative coordination lead /symptom manager	Human-led; AI supportive only	Override permitted at any time; escalate to multidisciplinary team for instability	Monitor access to symptom relief technologies across settings	Symptom burden; family satisfaction; goal-concordant care

AI, artificial intelligence.

### 3.1 Risk screening and diagnosis stage

In this phase, machines—such as multi-source risk prediction models and imaging-aided diagnostic systems—process large volumes of data to identify patterns, contribute to expanded screening capacity and may support earlier detection when integrated into appropriate clinical workflows (7, 28). Importantly, evidence for these potential benefits derives primarily from reader studies and modeled workflow simulations rather than from fully implemented population screening programs; consequently, findings should not be interpreted as definitive evidence of universal effectiveness in routine practice.

Within such workflows, the nurse’s role shifts from performing screening tasks to serving as a “connector” and “navigator” (28, 29). Key responsibilities include identifying high-risk individuals based on predictive outputs, providing tailored health education, preparing and counseling patients before examinations, and interpreting technical findings within the patient’s personal and psychosocial context (29, 30). This “Screening-Navigation” model combines the scale and speed of machine analysis with the individualized guidance and reassurance offered by nurses (12). In operational terms, the Screening–Navigation model refers to a structured workflow that combines algorithmic risk identification with nurse-led coordination of referral, education, and follow-up (31, 32). This model generally operates within organized screening programs with established diagnostic confirmation pathways and available navigation services (33).

### 3.2 Treatment decision-making and implementation stage

Here, machine systems—such as genomics-informed treatment recommenders—synthesize clinical evidence to suggest therapy options and forecast side effects (34, 35). The nurse acts as a “decision coach” and “holistic care coordinator,” integrating patient values, social contexts, and life goals into the decision-making process (36, 37). While machines present data-driven options, nurses address complex psychosocial needs—such as emotional distress or social isolation—and provide human-centered care during treatment delivery (38). This “Decision-Support” collaboration is intended to help balance clinical evidence with personal values, although outcomes depend on communication quality and contextual interpretation.

### 3.3 Rehabilitation and long-term survivorship stage

During rehabilitation and survivorship, wearable devices and digital platforms enable continuous monitoring, reminders, and education (39, 40). Nurses assume the role of “personalized health coaches” and “emotional supporters” (41, 42). They translate data from devices into tailored recovery plans, discern clinically relevant signals from false alarms, and offer empathetic support, hope, and help in finding meaning—functions that currently remain difficult to replicate through algorithmic systems alone (43). This “Monitoring-Coaching” dynamic may allow machines to assist with routine tracking while nurses manage complexity and provide relational care (40).

### 3.4 Advanced care and palliative/hospice stage

In end-of-life care, machines support comfort through precise symptom management devices, such as smart infusion pumps. The nurse becomes the “humanistic presence” and “primary comfort provider,” leading conversations on meaning, supporting families, and offering empathetic companionship that transcends technical intervention (44, 45). In this “Tool-Presence” model, technology can function as an instrument to enhance physical comfort, provided it aligns with patient goals and operates under clinician guidance. Meanwhile, nursing provides forms of human connection that are not presently reproducible by technological systems (46–48).

In summary, the evolving forms of human-machine collaboration across care stages reflect a strategic shift in nursing: from task execution toward roles that require integration of technology with psychosocial insight, relational support, and humanistic presence.

## 4 Key issues and challenges from a nursing science perspective

The integration of human-machine collaboration into breast cancer care presents several critical challenges that extend beyond operational effectiveness to touch the ethical and professional foundations of nursing. These issues demand comprehensive examination from a nursing science standpoint.

### 4.1 Reconfiguration of care relationship: from dyadic to triadic interaction

As technology becomes integral to care, the traditional nurse-patient relationship shifts to a triadic nurse-machine-patient dynamic (49). Health informatics scholarship further indicates that digital infrastructures do not merely assist clinical work; they actively reorganize how communication unfolds, redistribute professional attention, and influence how shared decisions are framed and negotiated (50, 51). This underscores that technology is not a neutral mediator but an active agent in care relationships (51, 52). Consequently, a primary challenge is to ensure that technology enhances rather than replaces the therapeutic human connection (53). Machines can process data and support decisions, but they cannot provide empathy, trust, or emotional presence (54). Therefore, nurses should consciously maintain the relational core of care by defining clear boundaries for technological tools, using them to facilitate—not obstruct—meaningful communication. For example, visualized data can help explain a diagnosis, but should not substitute for compassionate dialogue (55, 56).

### 4.2 Boundaries of clinical judgment: decision-making authority and critical digital thinking

When algorithmic recommendations conflict with clinical intuition or patient context, clarifying decision-making authority becomes essential. Clear guidelines should position technology as a supportive, not deterministic, tool—preserving nurses’ professional judgment and accountability (57). To minimize ambiguity and reduce automation bias, we propose a streamlined governance structure applicable across all stages of care (58, 59). Within this structure, ultimate decision-making authority remains with licensed clinicians (60). Any instance in which clinical judgment overrides an algorithmic recommendation should be comprehensively documented. This documentation should include the AI’s specific output, the clinician’s detailed rationale, relevant patient preferences, and the final action taken (59, 61, 62). This practice formally positions AI in a strictly advisory role while preserving professional accountability and upholding established standards of clinical practice (62).

Furthermore, mandatory escalation protocols are required in three specific scenarios: when the AI system indicates low confidence in its output, when it operates outside its validated scope, or when a disagreement between the clinician and the algorithm involves potentially high-risk outcomes (63–65). Such cases should be reviewed by a designated authority, such as a multidisciplinary team or a digital governance lead. This structured escalation ensures systematic oversight for high-stakes or uncertain situations.

Concurrently, nursing education should foster “critical digital thinking”: the ability to evaluate data sources, recognize algorithmic limitations, and interpret technological outputs within holistic patient care contexts (66, 67). This competency safeguards against overreliance and supports informed and individualized decision-making, and prepares clinicians to effectively navigate and document instances of human-AI disagreement. Detailed stage-specific override requirements and escalation pathways are summarized in **Supplementary Table 2**.

### 4.3 Ethics and equity: risks in technology-mediated care

Human-machine collaboration introduces distinct ethical risks. Algorithmic bias, rooted in unrepresentative training data, may worsen health disparities (68). Patient privacy and data ownership require transparent governance and consent processes (69). Additionally, excessive dependence on technology risks “de-skilling”—eroding nurses’ observational and assessment capacities (70). Proactive equity reviews and ethical oversight should therefore be embedded throughout technology development and implementation (71).

### 4.4 Valuing emotional labor: recognizing the human core of nursing

As machines take on information-based tasks, the relational and emotional aspects of nursing—such as providing comfort, facilitating difficult conversations, and building trust—become even more salient (72). These forms of “emotional labor” should be formally recognized and integrated into professional evaluations and institutional support structures (73). Although concepts such as emotional labor and critical digital thinking are discussed theoretically in this perspective article, they also provide clear directions for future practical application. Emotional labor can be assessed using established tools for measuring nursing workload and relational care. By safeguarding time and resources for relational care, healthcare systems can affirm the irreplaceable human contribution of nursing (74). Furthermore, critical digital thinking can be operationalized into measurable competencies within nursing education, simulation training, and ongoing professional development (75, 76). Clarifying these pathways helps connect conceptual insights with future empirical research. At present, there is insufficient consensus to support the implementation of a universal measurement instrument. We therefore propose the following candidate indicators for future evaluation: competency-based simulations of safe override procedures, documentation quality of clinical decisions, patient-reported relational experience metrics, and composite measures of staff wellbeing and cognitive workload (77–79). These proposed indicators do not constitute validated measurement standards. Rather, they should be regarded as preliminary constructs intended to guide subsequent empirical development, testing, and consensus-building efforts. As such, these indicators offer a concrete, albeit provisional, foundation for operationalizing and assessing both critical digital thinking and emotional labor within the proposed framework.

### 4.5 Digital literacy and education: competencies for future practice

To practice effectively alongside technology, nurses need structured digital competencies beyond technical proficiency (80). These include data literacy, the ability to critically assess digital tools, skills for interdisciplinary collaboration in digital environments, and the wisdom to balance technological and humanistic care (81, 82). Nursing curricula and professional development should evolve to cultivate these capacities, preparing nurses to lead, coordinate, and advocate within technology-enhanced care environments (82).

### 4.6 Safety and performance assurance

Responsible integration of AI into clinical care necessitates ongoing system surveillance that extends beyond point-of-care decision-making. A robust assurance framework should systematically incorporate the following elements: (1) Scheduled performance monitoring at established intervals (83) (2) Proactive detection of dataset or workflow changes that would trigger formal re-validation (84) (3) Routine maintenance of audit logs to provide a transparent record of how AI outputs influenced clinical actions (85) (4) A clear, accessible mechanism for reporting any suspected harm or system malfunction (83) and (5) Periodic equity assessments to identify and mitigate performance disparities across relevant patient subgroups (86). These structured lifecycle practices ensure that nursing leadership aligns with international governance standards for trustworthy AI, thereby promoting a sustained commitment to patient safety, clinical efficacy, and health equity.

## 5 Toward a synergistically enhanced nursing practice paradigm

Building upon the analysis of specific practices and core challenges, this perspective article proposes a fundamental shift toward a Synergistically Enhanced Nursing Practice Paradigm. This approach seeks to reconceptualize the human-machine relationship, positioning technology as an integrated extension of nursing expertise rather than an external tool. Its ultimate goal is to enhance the overall quality of patient care.

### 5.1 Conceptual positioning: from tool to cognitive partner

Within this paradigm, intelligent systems—particularly those driven by AI—function not as passive instruments but as cognitive extensions that augment nursing judgment (87). Their primary value lies in enhancing nurses’ capacities for risk assessment, condition monitoring, and evidence integration through advanced data processing, rather than replacing clinical decision-making or relational care (88). The goal, therefore, is not automation but augmented practice, where human expertise and machine capability may combine to support more precise, timely, and person-centered outcomes (89).

### 5.2 System design principles: a nursing-centered approach

To effectively implement this paradigm, the design of human-technology systems should be fundamentally guided by nursing practice. This requires adherence to four core principles. First, workflow integration demands that technological tools align with and enhance existing clinical processes, reducing rather than increasing operational burden (90). Second, the principle of professional autonomy should be explicitly safeguarded, ensuring nurses retain ultimate interpretive authority over algorithmic outputs and the right to override system recommendations based on clinical judgment (10). Third, transparency and explainability are essential for building trust; system interfaces should be intuitive, and decision logic should be traceable and comprehensible to nurse users (91). The necessary depth of algorithmic explainability should be calibrated to the level of clinical risk involved (92). Furthermore, explainability, while necessary, is insufficient to guarantee safe clinical integration (93). Its effectiveness depends on being embedded within a broader framework that includes comprehensive workforce training, robust governance protocols, and ongoing system evaluation (94). Finally, systems should be designed with relationship enhancement as a primary goal, ensuring technology facilitates rather than impedes meaningful nurse-patient interaction—for example, by automating administrative tasks to create space for interpersonal care (95).

### 5.3 Framework for practice: operationalizing the paradigm

Translating these principles into sustainable practice requires the development of concrete institutional and educational frameworks. A foundational step involves establishing stage-specific collaboration protocols that clearly delineate human and machine roles, data flow, and communication standards at each phase of the care pathway (10). Concurrently, healthcare organizations should implement structured validation processes wherein nursing and clinical experts critically evaluate algorithmic tools for contextual relevance, safety, and bias before integration into care plans (95). Furthermore, to ensure competency development and quality assurance, healthcare systems should integrate human-technology collaboration metrics into formal nursing quality indicators and embed related competencies—including digital literacy, ethical reasoning, and collaborative decision-making—into mandatory continuing education and specialty certification programs (96). Importantly, the operationalization of this framework will differ substantively across healthcare systems, contingent upon their existing digital infrastructure, workforce capacity, and regulatory development (97). In resource-limited settings, human–AI collaboration may logically prioritize foundational decision support and care coordination functions over more advanced automation (98). Recognizing these contextual variations is fundamental to avoiding a one-size-fits-all approach and to ensuring that technology-enhanced nursing practice advances equitably, remains adaptable, and sustains relevance across diverse global contexts (99). This multifaceted framework ensures the paradigm is operationalized, evaluated, and refined within real-world clinical environments.

### 5.4 Implementing the paradigm: a tiered feasibility approach

To ensure practical application, the implementation of AI in clinical settings can be structured into two feasibility tiers, each reflecting different levels of organizational readiness and technical capacity (100). The minimum viable tier corresponds to environments with constrained interoperability, limited workforce expertise, and foundational digital infrastructure (83, 101). In such contexts, AI applications are best focused on circumscribed, assistive roles—such as generating structured risk alerts, supporting documentation, and enabling basic monitoring—while maintaining rigorous downtime protocols that permit immediate reversion to conventional care processes without dependency on AI (83, 100, 102, 103).

The advanced tier applies to organizations with integrated data ecosystems, mature technical infrastructure, and dedicated analytical support. This enables the deployment of more sophisticated AI functionalities, including adaptive learning systems, predictive analytics, and tailored clinical or operational personalization (104). Crucially, both tiers require explicit operational definitions across four domains: staffing roles and competencies, necessary training regimens, well-specified fallback workflows, and a focused evaluation plan to measure impact, safety, and iterative improvement (105). This tiered framework supports a structured, evidence-based approach to AI integration, aligning technological deployment with institutional capabilities to promote scalable and sustainable implementation (105).

## 6 Summary and future directions

This study establishes that the effectiveness of human-machine collaboration in personalized breast cancer care is fundamentally contingent on aligning technological development with the core tenets of nursing science: preserving human dignity, fostering therapeutic relationships, and attending to the whole person. Any technological integration should therefore serve to strengthen, not undermine, the humanistic foundations of care. It is important to emphasize that the integrative framework proposed in this perspective is conceptual in nature and has not yet been empirically tested in clinical settings. As a perspective grounded in nursing science, its primary aim is to offer theoretical clarification, professional positioning, and strategic direction rather than immediate generalizable evidence.

Future research should prioritize three interconnected areas. First, comprehensive empirical research is imperative to evaluate the feasibility, effectiveness, and patient-centered outcomes of human–AI collaborative nursing models. This work should adopt rigorous mixed-methods frameworks, implementation science approaches, and longitudinal designs to examine both clinical processes and experiential impacts. Second, AI governance in nursing requires ethically informed guidelines derived from nursing theory itself, rather than imported from other disciplines. Third, interventions should be designed to cultivate nursing leadership in digital health, enhancing both technical fluency and adaptive implementation skills.

Ultimately, the nursing profession should move beyond a passive adoption role to actively shape the co-design, implementation, and critical assessment of care technologies. By assuming this proactive stance, nurses can play a decisive role in helping technological advances illuminate—rather than obscure—the relational and empathetic core of care. In doing so, they will help forge a future for breast cancer care that is not only more precise and data-informed, but also more profoundly human.

## Data Availability

The original contributions presented in the study are included in the article/**Supplementary Material**. Further inquiries can be directed to the corresponding author.
