# Supplementary material for: A nursing perspective on human-AI collaboration in personalized breast cancer care pathways
Source: Front Oncol. 2026 Mar 11;16:1784401. doi: 10.3389/fonc.2026.1784401 (PMC13012981; doi:10.3389/fonc.2026.1784401)
Supplement: Supplementary file 1 [file Table1.docx]

**Supplementary Table 1.Functional Categorization of Human-AI Collaboration and Its Application Across Stages of Personalized Breast Cancer Care Pathways**

| Collaboration Function Category | Definition & Typical Tasks | Applicable Care Stages | Nurse Role &  Responsibilities | Key Safety & Control Mechanisms |
| --- | --- | --- | --- | --- |
| Decision Support | AI provides evidence-based treatment recommendations, risk predictions, or genomic interpretations. Nurses make final decisions by integrating clinical judgment and patient values. | Treatment Decision-Making,  Rehabilitation Planning | Decision Coach,  Holistic Care  Coordinator | -Final decision authority rests with the nurse -Override documentation requirements (AI output, clinical rationale, patient preference) -Multidisciplinary review for high-risk decisions |
| Triage Assistance | AI assists in identifying high-risk or priority cases (e.g., imaging abnormalities, symptom deterioration).  Nurses confirm, prioritize, and follow up. | Screening & Diagnosis,  Rehabilitation Monitoring | Navigator, Connector | -Mandatory human review mechanism -Transparency of triage rules -Incident reporting system for missed/ false alerts |
| Monitoring &  Alerting | AI continuously tracks symptoms, adherence, or risk changes via wearables or EHRs. Nurses interpret, intervene, and provide emotional support. | Rehabilitation & Long-Term  Survivorship, Palliative/Hospice Care | Personalized Health  Coach, Emotional  Supporter | -Alarm fatigue management -Adjustable alert thresholds -Human confirmation required before intervention |
| Documentation Assistance | AI assists in generating nursing notes, discharge summaries, or follow-up plans. Nurses review, correct, and personalize the content. | All Stages (especially  Treatment & Rehabilitation) | Information Integrator,  Communication Coordinator | -Documentation audit and version control -Final nurse sign-off required -Patient informed consent mechanisms |
| Patient Support  Tools | AI provides health education content, medication reminders, appointment navigation, or psychosocial support resources. Nurses offer personalized explanations and emotional supplementation. | Screening Education,  Treatment Adherence Support,  Rehabilitation Guidance | Educator, Supporter,  Navigator | -Clinical review of content -Privacy protection for patient usage data -Regular nurse follow-up and outcome evaluation |
| Automated Execution | AI executes standardized tasks under defined rules (e.g., dose calculation, infusion pump control). Nurses supervise operations and handle exceptions. | Treatment Implementation,  Symptom Management  (e.g., analgesia) | Supervisor, Safety  Gatekeeper | -Automatic system pause on detection of anomalies -Human review of critical parameters - Regular device calibration and log audits |

**Abbreviations**: AI, Artificial Intelligence; EHRs, Electronic Health Records.
