# Supplementary material for: A nursing perspective on human-AI collaboration in personalized breast cancer care pathways
Source: Front Oncol. 2026 Mar 11;16:1784401. doi: 10.3389/fonc.2026.1784401 (PMC13012981; doi:10.3389/fonc.2026.1784401)
Supplement: Supplementary file 2 [file Table2.docx]

**Supplementary Table 2. Override and Escalation Logic by Care Pathway Stage**

| **Care Stage** | **Who Decides** | **Override Documentation**  **Required** | **Escalation Triggers** | **Escalation Reviewer** |
| --- | --- | --- | --- | --- |
| Screening &  Diagnosis | Nurse (with radiologist confirmation for  high-risk cases) | AI risk score, clinical rationale, patient preference, final decision | •Discrepancy between AI output and  radiologist/nurse assessment in high-risk case •AI flags “low confidence” or “out of scope”  case •Patient declines recommended follow-up | Radiology lead or  designated nurse supervisor |
| Treatment Decision-  Making | Nurse in partnership with patient (supported by oncologist) | AI-recommended options, patient values/goals, clinical justification, chosen plan | •AI-ranked options conflict with patient’s stated priorities •Disagreement between AI suggestion and  multidisciplinary team recommendation •Ethical dilemma (e.g., resource allocation, trial  eligibility) | Tumor board or clinical  ethics committee |
| Rehabilitation &  Survivorship | Nurse (as health coach) | AI-generated alert/adherence data, patient-reported status, action taken | •Persistent symptom alerts without clear cause •AI fails to detect deterioration (false negative) •Patient disengagement from digital monitoring | Rehabilitation coordinator  or digital health nurse lead |
| Palliative &  Hospice Care | Nurse (as primary  comfort provider) | AI-suggested symptom management, comfort assessment, patient/family input | •AI-driven intervention contradicts  patient/family wishes for comfort-focused care •System alert fatigue leading to missed critical changes •Spiritual/existential distress not addressed by AI support | Palliative care team lead or  hospice medical director |

**Abbreviations**: AI, Artificial Intelligence.
